# Food Advertisement and Marketing Policies Aimed at Reducing Childhood Obesity: A Review of Existing Regulations in High-Income Countries

**DOI:** 10.3389/phrs.2024.1607103

**Published:** 2024-12-20

**Authors:** Rida Khan, L. Suzanne Suggs, Afifa Tanweer, Gábor Bányai

**Affiliations:** ^1^ Institute of Health Sciences, Faculty of Health Sciences, University of Debrecen, Debrecen, Hajdu-Bihar, Hungary; ^2^ Institute of Communication and Public Policy, Faculty of Communication, Culture and Society, University of Lugano, Lugano, Switzerland; ^3^ Department of Nutrition and Dietetics, School of Health Sciences, University of Management and Technology, Lahore, Punjab, Pakistan; ^4^ Institute of Health Economics and Management, Faculty of Economics and Business, University of Debrecen, Debrecen, Hajdu-Bihar, Hungary

**Keywords:** food, advertisement, marketing, childhood, obesity

## Abstract

**Objectives:**

To identify and evaluate the difference between voluntary and mandatory food marketing policies and regulations targeting childhood obesity and to study the role of media, the food industry, and private associations in implementing such policies.

**Methods:**

A review of policies and legislation about unhealthy food marketing was conducted by searching and extracting relevant grey literature from the websites of international health agencies, food marketing pledge databases, GINA, and NOURISHING policy databases. Statutory laws and self-regulations of high-income countries were compared with each other and with the WHO recommendations.

**Results:**

Regulations differ regarding target audience, nutrient profiling, communication media, and marketing techniques. To date, no country has implemented comprehensive regulations restricting all forms of unhealthy food marketing. Statutory laws are more meticulous and rigorous than self-regulatory policies.

**Conclusion:**

The goal of reducing childhood obesity through restrictions on unhealthy food advertising has not been met. While not welcomed by all actors, mandatory regulations may be more effective than voluntary measures in reaching this goal. A system for monitoring adherence to regulations and providing both incentives and penalties for violations is warranted.

## Introduction

Childhood obesity is a pressing health, policy, and economic issue and its prevalence is increasing across the world. Children with obesity are more likely to become adults with obesity and have an increased risk of premature mortality [1]. Almost 2.6 million people die annually due to overweight and obesity [2]. Obesity results from a combination of multiple exposure factors in childhood to obesogenic environments [3]. Lifestyle factors including unhealthy diet and lack of physical activity along with environmental risk factors are the leading cause of childhood obesity [4]. Food marketing is often cited as a contributing factor to obesity [5]. Food marketing is purposively directed toward children, and they cannot distinguish advertising puffery from truth. As they grow older food choices solidify, and it becomes difficult to change preferences. Different promotion techniques in food marketing increase awareness, liking, and intention to consume foods [6] and nearly all the food marketing promotions are unhealthy. Food advertisements focus on taste, enjoyment, and gratification, making self-restraint difficult and stimulating hunger or thoughts of food. Attractive food packaging and marketing at sale points can trigger unplanned purchases [7].

A rapid review by Coleman et al (2022) explained the temporality, dose-response relationship, and biological mechanism to demonstrate the causal relationship between exposure to food marketing and childhood obesity [8]. Several studies have also examined the potential link between food marketing and childhood obesity. Montaña et al (2019), performed a study in Spain and found that low nutritional value product advertisements activate children to consume these products by showing associated positive emotions and experiences [9]. Richmond et al (2020), found that food and beverage advertising on public transportation is an unavoidable experience for children. Exposure to unhealthy food advertising on school trips can influence purchase decisions and increase the incidence of childhood obesity [10]. Research done in South Africa reported that unhealthy food advertisement was four times higher than the healthy during child and family television viewing time. Cartoons, celebrities, brand images, and health claims were more frequent in unhealthy versus healthy food advertisements [11].

Therefore, restricting unhealthy food advertisements to children can prove beneficial in lowering childhood obesity. Mytton et al (2020), estimated that restriction of unhealthy food advertising on television in the UK (United Kingdom) between 05.30–21.00 would make a significant contribution to decreasing childhood obesity [12]. Since the 1970s health agencies have issued guidelines to regulate food marketing to reduce childhood obesity. Many sectors attempt to establish rules and regulations about the advertising of food and beverages but gaps still exist [13]. Research has shown that the food industry’s pledges to market responsibly are ineffective in reducing the prevalence of unhealthy food marketing to children [14, 15]. Industries show an initial commitment to respecting regulations but persistently violate them in their advertising strategies [16].

This review aims to identify policies and guidelines for food marketing to children and evaluate the difference between voluntary and mandatory efforts toward lowering childhood obesity through food marketing restrictions. The secondary aim was to study the role of media, industries, and associations in regulating and implementing such policies.

## Methods

### Overview and Search Strategy

A grey literature review was conducted iteratively using targeted websites and databases. The preliminary search strategy included the following keywords derived from the title of the review in grey literature database^1^ and Google search engine^2^: “childhood,” “obesity,” “overweight,” “food,” “beverage,” “marketing,” “advertisement,” “laws,” “regulations,” “guidelines,” “recommendations,” “self-regulation.” This search led to the identification of the following major sources of the relevant grey literature: (i) websites of international health agencies; (ii) University of Connecticut Rudd Center for Food Policy and Health, Pledge Database on Food Marketing to Children Worldwide [17]; (iii) World Health Organization (WHO) Global Database on the Implementation of Nutrition Action (GINA) [18]; (iv) World Cancer Research Fund NOURISHING policy database [19]; (v) government websites for country specific statutory jurisdictions and self-regulation. Guidelines, recommendations, laws, self-regulation, and voluntary pledges targeting food and beverage marketing to children in specific and to all the population including children in general were reviewed.

### Eligibility Criteria

The latest version of full-text documents published by the government or non-government organizations between 2000–2022 (except for the statutory laws published and updated before the year 2000) available in the English language were included. Documents explicitly covering guidelines to prevent obesity or overweight by restricting food and beverage marketing and advertisement were selected.

Documents related to the marketing of breastmilk substitutes or formula milk, alcoholic beverages, and school lunch programs were excluded. Reports covering exclusively one form of marketing (e.g., outdoor media and schools) were excluded. Since, the main aim of this review was to identify various legislations and not to describe the impact of those legislations upon implementation, peer-reviewed literature and newspaper/magazine articles describing the latter were excluded from the study.

### Literature Selection and Data Charting

First, the guidelines, policy briefs, and action plans of the global custodians including WHO, United Nations International Children’s Emergency Fund (UNICEF), and the European Commission were retrieved from their respective websites and reviewed. Updated documents related to diet, physical activity, food and non-alcoholic beverage marketing and advertisement, childhood obesity, and overweight between the years 2000–2022 were accessed for relevance and included in the review (Table 1).

**TABLE 1:** Food marketing guidelines of global public health and child health agencies (Global, 2000–2022).

Custodian	Title	Year	Target audience	Objective	Recommendation	Actions and accountability
WHO	WHO Global Strategy on Diet, Physical Activity and Health [20]	2004	All age groups	Reducing the burden of noncommunicable diseases through promoting healthy diets and physical activity	Government should provide accurate information and discourage the marketing, sponsorship and promotion of unhealthy food and beverages to children	Review current marketing practices and exercise responsible marketing to children Take actions to minimize the harmful impact of food marketing
WHO	Set of recommendations on the marketing of food and non-alcoholic beverages to children [21]	2010	Not specified	Reduce the impact, exposure, and power of unhealthy food marketing on children	Member states should consider stepwise or comprehensive approach to achieve objectives Governments should be key stakeholders and set clear definition of components for standard implementation Settings of children gathering should be free of unhealthy food marketing	Policy implementation can be through statutory regulation and/or industry led self-regulation and/or voluntary initiatives Cooperation between member states to decrease the impact of cross border marketing Definition of sanctions and procedure of complaint registration should be included
WHO	Global Nutrition Targets 2025: Childhood overweight Policy Brief [22]	2014	<5 years of age	No increase in childhood overweight	Address the exposure of unhealthy food marketing to children	Regional offices should develop nutrient profiles Government should develop a timetable for regulating food marketing to children Impose tax on the marketing of unhealthy food and beverages to children Remove the unhealthy food and beverage vending machines in schools Remove unhealthy food and beverages at shop checkouts
European Commission	EU Action Plan on Childhood Obesity 2014–2020 [23]	2014	<18 years of age	Reduce the increase in obesity and overweight in children and young adults by year 2020	Restrict marketing and advertisement of HFSS to children	Make the schools free from the advertisement of unhealthy food Develop appropriate nutrition criteria for food marketing to children Set recommendation for food marketing via broadcast and non-broadcast media (for children <12 years of age) Encourage media industry to set strict code of conduct for audio visual marketing of food to children
WHO	Report of the commission on ending childhood obesity [24]	2016	<18 years of age	Address the obesogenic environment through the control of critical elements	Implement programs that increase the intake of healthy food and decrease the intake of unhealthy food and beverages Develop nutrient profile to distinguish unhealthy foods and beverages Implement recommendations on food marketing to decrease exposure to and power of unhealthy food and beverage marketing Develop cooperation among member nations to decrease the cross-border unhealthy food and beverage marketing	Government use their regulatory power to implement recommendations on marketing Develop a framework to implement recommendations among member nations
WHO	Global action plan on physical activity 2018–2030: more active people for a healthier world [25]	2018	All age groups	Create active societies, environments, and people	Implement regular participation in physical activities at public spaces Provide equitable access to safe, good quality and age friendly public spaces for physical activity Promote the provision and participation in physical activities at school	Partner with stakeholders to promote physical activity in open public spaces, parks, sport facilities before and after school if promotion of product/brand follows WHO marketing recommendations to children Implement marketing restriction on unhealthy food and beverages in open public spaces, parks, schools, and sport facilities
UNICEF	Prevention of overweight and obesity in children and adolescent: Programming guidance [26]	2019	<18 years of age	Support government efforts in developing polices to prevent overweight in children	Take *de novo* double duty actions in regulating the unhealthy food marketing to children	Conduct situational analysis of the marketing regulations in place Review marketing of unhealthy food and beverages to children Improve the food environment through the implementation of WHO marketing recommendations
UNICEF	Marketing of unhealthy foods and non-alcoholic beverages to children: Policy Brief [27]	2021	<18 years of age	Protect the children from unhealthy food marketing	Marketing restrictions should protect the children of all ages, apply to full range of channels and cover promotion and sponsorship Concept of food marketing should be broader and more inclusive of time and placement Nutrient profile model should determine the unhealthy food Stricter implementation strategies are needed	Statutory legislation is superior to industry led self-regulation Government is responsible to set country specific goals, lead the process with industrial collaboration, monitor effectiveness and allocate budget for implementation and policy education campaigns
UNICEF	Protecting children from the harmful impact of food marketing: Policy brief [28]	2022	<18 years of age	Provide policy options to protect children from harmful impact of food marketing	Eliminate all forms of unhealthy food marketing to children Eliminate all forms of food marketing to children Eliminate all forms of marketing to children	Comprehensive approach is superior to stepwise approach

According to the updated list of World Bank 2022, high-income countries having a Gross National Income *per capita* of $13,205 or more [29] were selected for the next part of the review.

The GINA database is a global repository for country-specific guidelines related to the nutrition [18]. All high-income countries were searched individually on this database for “voluntary codes or measures relevant to nutrition” and “legislation relevant to nutrition.” After checking the eligibility criteria, government websites of selected high-income countries that were found to have either statutory laws or self-regulations for food marketing were searched for the latest documents of the selected policies and laws. The NOURISHING database provides implemented policies to promote healthy diets and reduce obesity worldwide [19] and it was searched for the policies of food environments that restrict food advertising and commercial promotion. Sub-policy area searches included mandatory requirements, voluntary agreements, government-supported self-regulation, and voluntary pledges. Policies from the selected high-income countries were searched on the relevant government websites for the latest documents. Statutory laws and self-regulations of the countries were compared to each other and with the WHO recommendations based on the target audience, nutrient profiling, communication media, and marketing technique covered (Tables 2, 3).

**TABLE 2:** Self-regulatory guidelines for food marketing in high-income countries (Global, 2000–2022).

Country	Authority	Title	Year	Target audience	Media covered	Recommendations	Food classification	Shortcomings
England	Committee of Advertising Practice (CAP)	The UK Code of Non-broadcast Advertising and Direct and Promotional Marketing [30]	2010	<16 years of age	Print media, emailing, posters, paid for space, paid for search listing, price comparison websites, Bluetooth, and web gadgets	Marketing should not encourage unhealthy eating practices and create a pressure to purchase Marketing of HFSS to children should not include promotional offers and licensed characters No medium of HFSS advertisement should be used if >25% audience is <16 years of age	Department of Health nutrient profiling model [31]	Does not cover product packaging and point of sale
Ireland	Advertising Standard Authority for Ireland	Manual of Advertising Self-Regulation [32]	2007	<18 years of age	Television, radio, cinema, print media, emailing, posters	Food advertisement should not encourage unhealthy eating, it should not mislead the social, physical, and psychological benefit of the product consumption and snacks should not be depicted as meal	No criteria; applies to all food products	Does not cover product packaging and point of sale
US	CARU, BBB	Self-Regulatory Program for Children’s Advertising [33]	2009	<12 years of age	Any medium including labelling and commercial websites	Amount of food advertised should not exceed the portion size Food advertisement should not discourage healthy eating and should depict the food for meal and snack as different	No criteria; applies to all food products	Product endorsement is allowed
Canada	Advertising Standards Canada (ASC)	The Broadcast Code for Advertising to Children [34]	2017	<12 years of age	Television, radio	Advertisement must not create pressure of buying Direct response techniques to invite for purchase are prohibited Promotion and sponsorship of the product advertised to children by famous characters and persons is prohibited Food marketing representing mealtime should mention the role of product in the balanced diet and snacks should not be depicted as a meal Quantity of food advertised should not be more than the portion size	No criteria; applies to all food products	Does not cover product packaging and point of sale Use of fictional characters and other persons used exclusively in advertisement are allowed
New Zealand	Advertising Standards Authority	Children and Young People’s Advertising Code [35]	2017	Children < 14 years of age Young people > 14 but < 18 years of age	Any medium	Sponsorship in the advertisement of occasional food and beverages targeted to children is prohibited Quantity of food in advertisement should not be more than the portion size Promotional offers must not encourage the excessive purchase and consumption of food and beverages	Food and beverage classification system [36]	Does not cover product packaging
Italy	Italian advertising standards authority	Code on commercial communication [37]	2004; updated 2021	<12 years of age	Any medium	Marketing of food and beverage should not encourage children to buy it Audiovisual marketing of HFSS should not emphasize the positive nutritional quality of the food There should be clear difference between program and advertisement Visual depiction of failure to consume the food will lead to social exclusion is prohibited		No nutrition profiling Does not define criteria for promotion and sponsorship of HFSS foods
Romania	Romanian Advertising Council	The Ethical code for food product advertising targeting children [38]	2015	<12 years of age; 35% audience is children	Television, internet, and print media	Advertisement should follow the EU pledge [39] It should not encourage the children to buy the product Sponsorship, promotion and use of licensed characters is prohibited if the product does not follow the minimum nutrition criteria and 50% audience is children Advertisement in primary school is prohibited Outdoor advertisement in the 50 km distance from school or children places is prohibited if does not follow the minimum nutrition criteria	EU Pledge Nutrition Criteria [40]	Does not cover product packaging and point of sale Higher percentage of children (50%) to restrict sponsorship, promotion, and use of licensed characters in HFSS food
Germany	German Advertising Council	Rules of Conduct of the German Advertising Council for all forms of commercial communication for food [updated] [41]	2021	<14 years of age	Any medium	Food advertisement should no solicitate for purchase to children, should not contain promotional offers and claim social or education success through the consumption of marketed product	No criteria; applies to all food products	
Norway	Food and Drink Industry Professional Practices Committee (MFU)	Guidelines for the marketing of food and drink aimed at children [42]	2007; updates 2019	<16 years of age	Website, social media, television broadcast before 21.00, advertisement in public space	Advertisement, sponsorship, and promotion of products included in the list of HFSS prepared by MFU to children and young people is prohibited	Product list [43]	Does not cover product packaging and point of sale
Spain	Ministry of Health	Spanish Self-regulatory Code for Food and Non-alcoholic Beverage Advertising Aimed at Children (the PAOS code) [44]	2005; updated 2012	<12 years of age for print and audiovisual; <15 years of age for online	Any medium	Advertisement should not promote unhealthy eating and lifestyle practices It should not exploit children’s credulity by giving misleading information and urge them to purchase the product It should avoid using famous persons popular among children Promotional offers should provide appropriate information	No criteria, applies to all commercial products	Does not cover product packing
Netherlands	Advertising Code Authority	The Dutch Advertising Code [45]	2017	<12 years of age; 25% audience is children	Any medium	Advertisement of food products associated with children’s program should not be broadcasted immediately after program Sponsorship and product promotion of such food items is not allowed Food advertisement should not suggest higher status and popularity with that food consumption	Nutrition criteria per portion size only available in Dutch	Does not cover product packaging and point of sale
Singapore	Advertising Standards Authority of Singapore	Children’s Code for Advertising Food and Beverage Products [46]	2015	<12 years of age	Any medium	Food advertisement should not encourage unhealthy eating and snacking, pressure to purchase Food promotion through premium offers and famous personalities is prohibited	Common Nutrition Criteria [47]	Exempt brand equity character Does not cover product packaging and point of sale

**TABLE 3:** Statutory laws to regulate food marketing in high-income countries. (Global, 1970–2022).

Country	Title	Year	Target audience	Media covered	Legislation	Food classification	Shortcomings
England	The UK Code of Broadcast Advertising (BCAP Code) [48]	2007	<16 years of age	Television, radio	Advertisement should not encourage practices harmful for children health, take advantage of children’s credulity, inexperience, and loyalty or directly exhort them into buying a product or service Promotional offers, licensed characters and celebrities should not be used in HFSS food advertisement Collection based promotions must not urge children to buy the bulk quantities of product	Department of Health nutrient profiling model [31]	Allow the use of advertiser created equity brand characters Apply only to radio and television Does not apply to print media and digital marketing at social media platforms
Ireland	Broadcasting Authority of Ireland Children’s Commercial Communications Code [49]	2013	<18 years of age; 50% audience is children	Television, radio, teleshopping	Commercial communication should not encourage unhealthy lifestyle, provide misleading information, include HFSS food in children’s programs Adverts are prohibited to contain nutritional claims, licensed characters, and promotional offers and use celebrities to promote HFSS food Fast food products should display message ‘should be eaten in moderation and as part of a balanced diet’	Department of Health nutrient profiling model [31]	Covers only broadcasting media and does not apply to digital and social media commercial
US	Children’s Television Act [50]	1990	<13 years of age	Television all time on-air	A featured character in a children’s program is prohibited to appear in a commercial during that program and a clear separation between the program and commercials is required	-	Only applies to television programs, does not apply to other broadcasting media, digital, social and print media No nutrition profiling No criteria for promotion and sponsorship of HFSS foods
Chile	The Law on Nutritional Composition of Food and its Advertising [51]	2016	<14 years of age; 20% audience is children	Any medium	Advertisement of HFSS is prohibited in school Advertisement and promotion of HFSS to children is prohibited by all means HFSS food advertisement on TV between 6:00 to 22:00 is prohibited	Phased implementation of threshold for high calorie and HFSS food [52]	Does not cover brand advertisement
Canada	Consumer Protection Act [53]	1980; updated 2012	<13 years of age	Radio, television, website, mobile phones, printed materials such as newspapers, magazines, and flyers, signage	Commercial advertising directed at children is prohibited Nature, manner, time, and placement of the advert determine whether it is directed at children	No criteria, applies to all commercial product	Advertisement in children’s magazine, children’s entertainment event, store displays, and packaging are exempted
Australia	Food and Beverages Advertising Code [54]	2021	<15 years of age	Any medium	Advertisement of occasional food and beverage must not be target children Sponsorship, prizes, awards and vouchers in the advertisement of occasional food and beverages targeted to children is prohibited Promotional offers must not encourage the excessive purchase and consumption of food and beverages	Nutrient Profiling Scoring Criterion [55]	Does not cover packaging
Finland	Consumer Protection Act [56]	1978; updated 2016	<18 years of age	Any medium	Marketing should not urge the children directly to buy product Marketing should be recognizable, not hidden It should not encourage unhealthy eating practices	No criteria, applies to all commercial product	Does not contain regulations for promotion and sponsorship of HFSS foods
Poland	The Broadcasting Act [57]	1992	Age not specified	Television, radio	Children’s program should not accompany advertisement of unhealthy foods	Criteria developed by Ministry of Health [58]	Apply only to radio and television advertisement Does not specify sponsorship and promotion rules
Norway	Broadcasting regulation [Amendment] [59]	1997; updated 2005	<18 years of age	Television, radio	Advertisement aimed at children is prohibited to use characters and figures featured in the programs for children in last 12 months Advertisement specifically directed at children is prohibited No advertisement before and after the children program	No criteria, applies to all commercial product	Apply only to radio and television advertisement
Hungary	The Hungarian code of advertising ethics [60]	2015	Not specified	Television, radio, printed media	Advertisement of food and beverages aimed at children should not apply famous personalities, mislead to have positive health, psychological and social effects and create a sense of urgency to buy	No criteria, applies to all commercial product	Children characters used in the food and beverage advertisement are only restricted if broadcasted in the same children program
Spain	Royal Decree on the regulation of food and beverage advertising aimed at children [DRAFT] [61]	2022	<16 years of age; 25% audience is children	Television, radio, social media, outdoor advertising, movie theaters, mobile application	Advertisement containing excessive quantity of HFSS food is restricted Use of fantasy element, influencers, and promotion offers to advertise HFSS food is prohibited HFSS food advertisement suggesting a replacement of meal, potential social and psychological benefit of product consumption and encourage children to ask other to buy for them is restricted No advertisement before and after the children program	Nutrition criteria developed by WHO for European region [62]	Draft waiting for approval and enforcement
Portugal	Advertising Code (14th amendment) [63]	2019	<16 years of age; 25% audience is children	Television, radio, movies, print media, internet, and social media	Advertising of HFSS and high calorie food to children is prohibited Food advertisement at school and playground and within 100-m radius is prohibited Advertising of HFSS and high calorie food should not promote excessive consumption, create a sense of urgency and benefit of exclusive consumption No advertisement before and after the children program	Nutrition criteria developed by WHO for European region [62]	Does not specify sponsorship and promotion rules
Sweden	The Swedish Radio and Television Act 1990 [64]	2010 Revised	<12 years of age	Television, radio	Commercial advertisement should not capture the attention of children Commercials should not occur immediately before and after children’s program Commercial advertisement should not contain characters from the children’s program	No criteria, applies to all commercial product	Apply only to radio and television advertisement
Malta	Broadcasting Code for the Protection of Minors [65]	2010	<18 years of age	Television, radio	Marketing of confectionery and snacks should not suggest that these products can be substituted for meals Advertisement should not encourage the children to eat frequently throughout the day	No criteria, applies to all commercial product	Applies to broadcast media only Encourage the media service provider to develop criteria for HFSS foods
Romania	The Code of Audiovisual Content Regulations [66]	2013	<12 years of age; 35% audience is children	Television, radio	Advertising directed to children should not contain children, cartoons, celebrities, and doctors Food advertisement should not discourage the consumption of fruits, vegetables, and natural products Health information messaging is mandatory between 6 am and 10 pm	No criteria, applies to all commercial product	Applies to broadcast media only Encourage the media service provider to develop criteria for HFSS foods
South Korea	Special Act on Safety Management of Children’s Dietary Life [67]	2010	<18 years of age	Television, radio, internet	Advertisement of unhealthy food along with free toy promotion to children is prohibited Advertising of unhealthy food is banned between 5 and 7 pm and during children show	Nutrition criteria defined by Korean Food and Drug Administration [68]	Does not cover wide range of media

The University of Connecticut Rudd Center for Food Policy and Health, Pledge Database on Food Marketing to Children provides private sector voluntary commitments [17] and it was used to identify authorities overseeing the food and beverage industries in different regions of the world. Councils and associations in high-income countries including the Council of Better Business Bureau (BBB) of the US and Canada, the International Council of Beverages Associations, the International Food and Beverage Alliance, the Union of European Soft Drinks Associations, Australian Food and Grocery Council, Australian Beverage Council, and Gulf Cooperation Council were selected. Pledges of European countries were excluded as they followed the previously described recommendation of the European Commission. Council websites were explored to retrieve the details of voluntary pledges of member industries (Table 4).

**TABLE 4:** Marketing pledges of food and beverage associations and the media industry. (Global, 2000–2022).

Authority	Title	Year	Target audience	Marketing pledge	Food classification	Number of signatories; implementation
International Food and Beverage Alliance	Global Responsible Marketing Policy [69]	2011; updated 2021	<13 years of age; 35% of audience is children	Food product not following the Common Nutrition Criteria should not be advertised to children through TV, radio, print, internet, cinema, influencers, licensed characters, and product placement No food advertisement in primary school, early childhood education and care institution	Common Nutrition Criteria [70]	11; Mandatory for all members, third party monitoring for compliance
International Council of Beverages Associations	Guidelines on Marketing to Children [71]	2009; updated 2015	<13 years of age; > 35% audience is children	Only advertise 100% fruit juice, water, and milk-based drink to children through TV, radio, print, internet, cinema, and product placement No food advertisement in elementary and middle school		10; Voluntary participation
	EU Pledge [39]	2007; update 2021	<13 years of age; 30% audience is children	Food products not fulfilling the EU Pledge Nutrition Criteria should not be advertised to children through TV, radio, print, internet, cinema, games, influencers, licensed characters, and product placement No food advertisement in primary school	EU Pledge Nutrition Criteria [40]	23; Voluntary participation
Union of European Soft Drinks Associations	Responsible Marketing and Advertising [72]	2006; updated 2021	<13 years of age; 30% audience is children	No advertisement of any beverage to children through TV, radio, print, online and cinemas No direct appeal to children in marketing		20; Voluntary participation
BBB	CFBAI [73]	2007; update 2022	<13 years of age; 25%–30% audience is children	Food products not following the Uniform Nutrition Criteria should not be advertised to children through TV, radio, print, internet, influencers, licensed characters, and product placement No food advertisement in elementary school	Uniform Nutrition Criteria [74]	20; Voluntary participation
Advertising Standards Canada (ASC)	CFBAI [75]	2007; update 2015	<12 years of age; >35% audience is children	Food products not fulfilling the Uniform Nutrition Criteria should not be marketed to children through TV, radio, print, internet, video games, DVDs, influencers, licensed characters, and product placement No food advertisement in elementary school	Uniform Nutrition Criteria [76]	16; Voluntary participation
Australian Beverage Council	Marketing and Advertising Initiatives [77]	2006	<15 years of age; 50% audience is children	No advertisement of sugar sweetened beverages to children, in primary schools and children’s program Encourage drinking bottled water, juice without added sugar and flavored milk to children		90% of the beverage industries in Australia; Voluntary participation
Australian Food and Grocery Council	Quick Service Restaurant Pledge [78]	2009	<14 years of age	Foods that do not comply to the nutrition criteria should not be marketed to children through product placement and promotion No food marketing in primary school, preschool and day care centers	Nutrition criteria defined to meals served in restaurants	7; Voluntary participation
Australian Food and Grocery Council	Responsible Children’s Marketing Initiative [79]	2009	<12 years of age; > 35% audience is children	Foods advertised to children should be healthy according to the Australian government standards Unhealthy food should not be marketed to children through product placement and promotion No food marketing in primary school, preschool and day care centers	Australian government standards to determine healthy foods; signatories make own nutrition criteria	17; Voluntary participation
Gulf Cooperation Council food and beverage alliance	The Responsible Food and Beverage Marketing to Children [80]	2010	<12 years of age; 35% audience is children	Food products not following the nutrition criteria set by EU pledge should not be marketed to children through TV, radio, print, online and cinemas	EU Pledge Nutrition Criteria [40]	7; Voluntary participation

Food marketing recommendations by the national agencies of the United States (US) were also reviewed and are given in the Supplementary Table S1.

A summary of the search strategy and literature selection is given in the Supplementary Figure S1.

## Results

### Global Agencies

In 1989, the United Nations (UN) Convention on the Rights of the Child (CRC) declared in Articles 17 and 18 that it is the duty of the government to develop “appropriate guidelines for the protection of the child from information and material injurious to his/her wellbeing” and assist the families in protecting their children [81]. In 2004, the 57th World Health Assembly (WHA) endorsed the Global Strategy of WHO to reduce the burden of noncommunicable diseases, which outlined that it is the responsibility of “government” to regulate food marketing to children [20].

In 2007, at the 60th WHA meeting, it was resolved that the WHO should develop a set of recommendations on the marketing of foods and beverages to children. Two extensive systematic reviews were conducted to report the evidence on the “extent, nature and effects of food marketing to children” [82]. Then a set of 12 recommendations with the goal to reduce the exposure and the power of marketing to children was endorsed at the 63rd WHA meeting in 2010. Exposure is the nature, audience, and frequency of adverts whereas, power is the design, content, and delivery of marketing message. Foods and beverages classified as unhealthy were those high in fat, sugar, and salt (HFSS). WHO suggested two approaches for the member states to achieve the policy goal [1]: a comprehensive approach restricting all HFSS food and beverage marketing to children [2]; a stepwise approach focusing on either exposure or power of marketing. For policy implementation, WHO proposed [1]: statutory regulations, legally requiring the implementation and compliance [2]; industry-led self-regulations, involving the compliance monitoring of industry-specified codes by an industry-sponsored body; and [3] co-regulation, codes approved by the government but monitoring role reserved for an industry-sponsored body [83]. Although this set of recommendations provided some novel strategies to the member states, it had a few areas of improvement which were progressively resolved in the future directions placed by WHO, EU, and UNICEF. These problems along with their identification and addressal by the same organizations are being presented here to aid the gradual building of the narrative and to retain the chronological order of our results section.

It was observed that the 2010 WHO recommendations lacked nutritional criteria for classifying food and beverages into the healthy and unhealthy category for children [21], thus, giving the liberty to the food industry to decide by themselves what is unhealthy. Later in 2014, WHO released a policy brief to achieve one of the six global nutrition targets, which is to ensure that there is no increase in childhood overweight by 2025 [22]. In this brief WHO directed the regional offices to develop the nutrient profile model following the international dietary guideline and befitting the regional context [22]. The same year, the European Union (EU) also launched its action plan on childhood obesity which suggested that member states should build nutrition criteria by 2016 [23]. Secondly, WHO recommendations suggested a stepwise approach of restricting the advertisement of specific food products through certain marketing channels with low efficiency in reducing the exposure to unhealthy food environments. Later in 2022, UNICEF declared its stance on the conflicting policy approaches and stated that a comprehensive approach is broad enough to cover all forms of marketing including cross border marketing, and hence can sufficiently protect the children [28]. Additionally, the insinuation to use industry-led self-regulation as an option [21] was soon realized as not a long-term solution [84]. Recently in 2021, UNICEF rectified that a government-led process with strict legislative objectives should be in place to solve this problem [27].

### Statutory Laws and Self-Regulation

**FIGURE 1:**
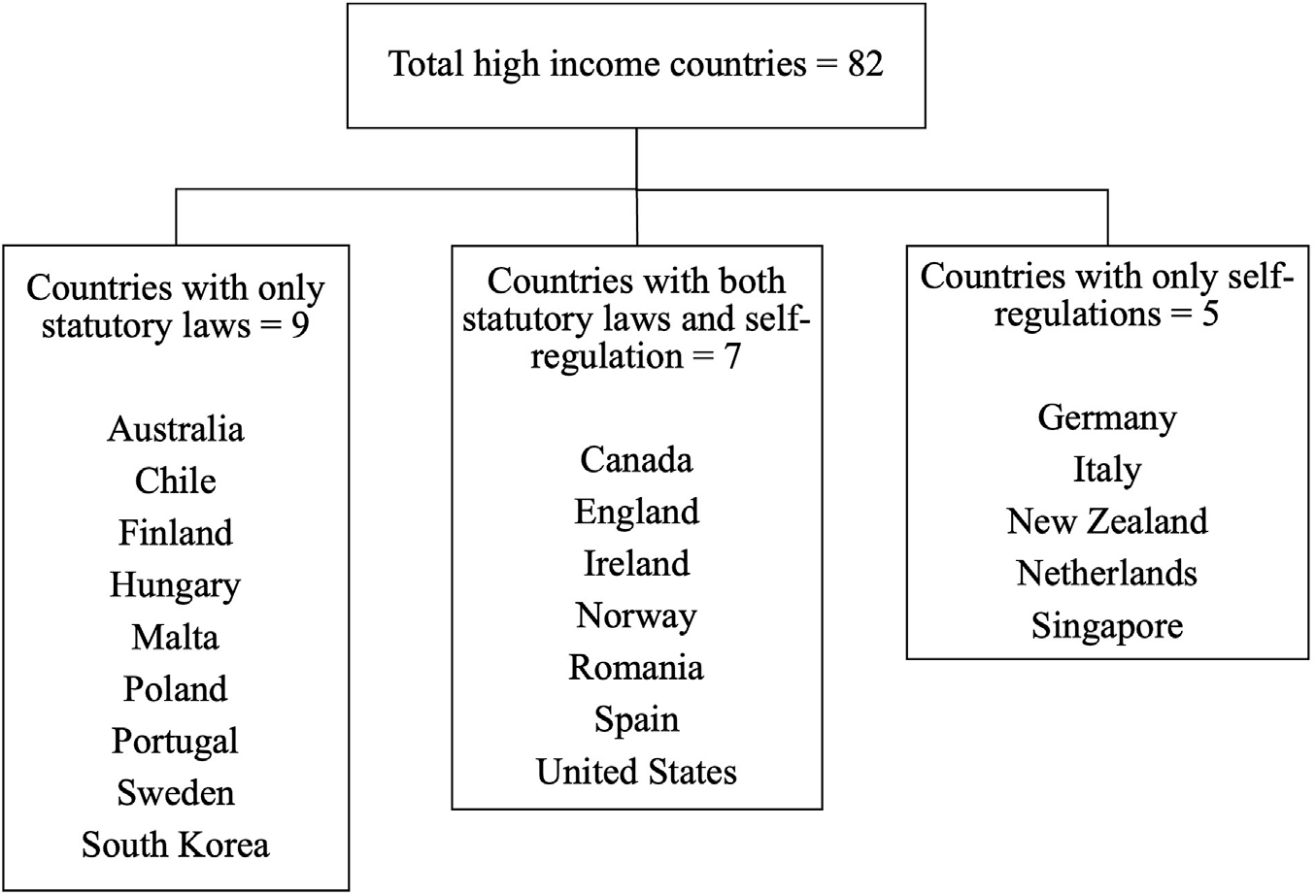
Summary of food marketing and advertisement regulations in high-income countries. (Global, 2000–2022).

16 high-income countries (England, Ireland, US, Chile, Canada, Australia, Finland, Poland, Norway, Hungary, Spain, Portugal, Sweden, Malta, Romania, South Korea) were found to have statutory laws (Table 3) and seven of them (England, Ireland, US, Canada, Romania, Norway, Spain) followed self-regulation at several points (Table 2). Five countries (New Zealand, Italy, Germany, Netherlands, Singapore) were found to have no statutory law, and the food advertisement is completely self-regulated (Figure 1). These legislations and self-regulations differ concerning the target audience, classification of unhealthy food, communication media, and marketing techniques covered.

#### Target Audience

Every country has its own definition of children-directed advertisement. These definitions consider the audience composition including the age of children and the percentage of children exposed to media communication.

Statutory advertisement restriction in Romania [66] and Sweden [64] is aimed at children less that 12 years old. In the US and Canada, they target children under 13 [53], in Chile under 14 [51], in Australia under 15 [54], and in England [48], Spain [61], and Portugal [63] they are aimed at children under 16 years of age. South Korea [67], Malta [65], Norway [59], Finland [56], and Ireland [49] have the highest age limit of under the age of 18. The threshold percentage of children exposed ranges from 20% to 50% [49, 51, 61, 63, 66]. Chile set the lowest threshold at 20% [51] which is considered adequate for protecting most children, but Ireland’s law applies only if 50% or more audience is under 18 years of age [49].

The age to be considered as children in the self-regulation by Netherlands [45], Spain [44], Romania [38], Italy [37], Canada [34], the US [33], and Singapore [46] is under 12 years old. For Germany [41] and New Zealand [35] that age is under 14 years and for Norway [42] and England [30] is younger than 16. Ireland is the only country that sets the age of under 18 years for the application of self-regulation [32]. Unlike statutory laws, self-regulations of all the countries except Romania (35%) [38] and the Netherlands (25%) [45] do not specify the percentage audience of children as a threshold.

#### Nutrient Profiling

Nutrient profiling defines the food as healthy or unhealthy and permitted or not permitted to market based on the amount of fat, salt, sugar, and calories that are present in the food per weight, volume, or portion size [83].

Statutory laws of eight out of sixteen countries (England, Ireland, Chile, Australia, Poland, Spain, Portugal, South Korea) follow nutrition criteria, whereas the rest of them have no criteria and restriction applies to all commercial products including food and beverages. The act by the broadcasting authorities of England and Ireland follows the nutrition criteria developed by the Department of Health [31]. Similarly, Poland also follows the criteria developed by their Ministry of Health [58]. In Europe, only Spain and Portugal follow the criteria developed by WHO for the European region [62]. On the other hand, Sweden, Malta, Romania, Hungary, Finland, and Norway have no criteria and restriction applies to all commercial products. South Korea uses the criteria developed by the Korean Food and Drug Administration [68]. Chile is the only country in the Americas with nutrition profiling in accordance with WHO and followed phased implementation of increasingly stringent criteria over the short course of 2 years from 2016 to 2018 [52].

Out of 12 countries that have self-regulation, half (England, New Zealand, Romania, Norway, Netherlands, Singapore) include nutritional criteria. England’s self-regulatory code for the non-broadcast media follows the same nutrition criteria as the statutory law. Romania follows the EU Pledge Nutrition Criteria [40] for self-regulation and the Common Nutrition Criteria [47] of Singapore is also consistent with the EU Pledge. New Zealand does not have any statutory law. Therefore, the food and beverage classification system [36] of the Advertising Standards Authority followed through self-regulation is much more detailed. Netherland’s nutrition criteria are based on just the portion size [45].

#### Media Covered

Most of the self-regulations apply to all media including television, radio, cinema, print media, emails, posters, commercial websites, internet, social media, and outdoor public spaces. In contrast, most statutory laws cover only broadcast media including just television and radio.

Half (England, Ireland, US, Poland, Norway, Sweden, Malta, Romania) out of 16 high-income countries only cover television and radio. The law in Chile [51], Australia [54], and Finland [56] cover all medium of commercial communications. England’s code for broadcasting media is statutory [48] and the code for non-broadcast media is self-regulatory [30]. Canada [53], Spain [61], and Portugal [63] restricted media covering television, radio, websites, mobile phones, printed media, and signage.

#### Marketing Techniques Covered

The power of commercial communications in appealing to the audience is suggested by different marketing techniques. The main point of almost every law is that advertisements should not encourage unhealthy eating practices or compromise the integrity of parents in making food decisions for children [30]. They should not create a sense of pressure and urgency to buy [44]. Food advertisements should not mislead children about the physiological and psychological benefits of consuming a particular food [32]. And they should not suggest that children will be more socially accepted among peers if they consume specific food products [60, 61, 63].

Few legislations were found to cover various marketing techniques, though. South Korea is the only country that prohibits the use of free toys for the promotion of unhealthy food products [67]. Some laws suggest that the advertisement should not take advantage of the loyalty of children and encourage them to pester their parents into buying excess amounts of food by offering collection-based promotions [35]. Hungary [60] and Romania [66] banned the use of cartoons and licensed characters in advertisements aimed at children but Norway only restricts them if the cartoon or personality appeared in a children’s program in the past year [59]. Germany [41] restricts the promotion in marketing of all food products, on the other hand, Sweden [64] and Canada [34] ban such promotional figures completely in all forms of commercial marketing communications. The Department of Health and Social Care in England also intends to ban the unlimited refill options [85].

Canada [53] and Norway [59] prohibit all forms of commercial marketing to children while other countries focus on HFSS foods and beverages. Chile [51], Ireland [49], and Portugal [63] prohibit the advertisement of HFSS foods to children in all forms, while England [30, 48] and Spain [61] just prohibit the promotion, and use of licensed characters and celebrities in HFSS food advertising. Ireland also prohibits the use of nutritional claims in HFSS food advertising [49]. The quantity of HFSS food in advertising is also considered; excessive quantity of HFSS in advertisement is prohibited and it should not exceed the portion size [61]. Commercials depicting unhealthy and occasional foods that could replace proper meals are prohibited [32, 61, 65]. Like HFSS foods, Australia also restricts the marketing and sponsorship of occasional foods to children [54]. Ireland and Romania also mandate that food marketing should be accompanied by healthy eating messages [49, 66]. According to the law in Finland [56] and Italy [37, 50], advertisements should be differentiated from programs. Broadcasting authorities of Poland, Sweden, and the Netherlands regulate that children’s programs should not be accompanied by advertisements for unhealthy foods [57], not immediately before and after the program [45, 59, 64].

### Limitations of Self-Regulation Vs. Statutory Laws

#### Self-Regulation

No self-regulation covers advertisements through food packaging and at the point of sale in retail stores except under the Rules of Conduct of the German Advertising Council [41]. Singapore’s Advertising Standards Authority allows the use of brand equity characters in food promotion [46]. Self-regulation in Romania has been found to be the weakest, as restrictions in sponsorship, promotion, and licensed characters only apply if more than 50% of the audience is under 12 years of age [38]. The Code on Commercial Communication by the Italian Advertising Standards Authority lacks the nutrition criteria to determine the HFSS and does not specify restrictions on promotion and sponsorship [37].

#### Statutory Laws

The major shortcoming is that they cover only the broadcast media whereas children on other media outlets like digital and social media are not protected. Brand equity characters are exempted [48]. Statutory laws in the majority of the countries do not cover product packaging and point of sale [54]. Most countries including Finland [56], Poland [57], and Portugal [63] do not cover the marketing techniques such as sponsorship and promotion. The Children’s Television Act of the US does not have nutrition profiling criteria [50]. Chile’s laws are considered the most expansive and covers product packaging and point of sale but do not cover brand advertisement [51]. Canada covers a wide range of media in the Consumer Protection Act but the advertisement in children’s magazines and entertainment events are exempted [53]. Although the Hungarian code of advertising ethics states that famous personalities are not allowed in children’s advertisements, yet children’s characters are only prohibited if the advertisement is broadcasted with children’s programs containing the same character [60]. Romania [66] and Malta [65] do not have any criteria to classify HFSS food and encourage media service providers to develop nutrition criteria by themselves, suggesting a sense of self-regulation. These laws do not cover special settings like educational institutions and special events like sports and entertainment activities.

### Food and Beverage Industries

Food and beverage industries were found to support the development of self-regulation and voluntary pledges to address the obesity crisis (Table 4). However, several weaknesses were observed in the industry-led self-regulatory pledges and are explained as follows:

• The nutrition criteria set by industries are weaker and more permissive than the criteria set by the WHO. The nutrition criteria are category-specific with a higher threshold for certain unhealthy nutrients in some food categories [40, 70, 74, 76]. The nutrition criteria developed by the Children’s Food and Beverage Advertising Initiative (CFBAI) in Canada [76] and US [74] set the limit of nutrients based on the “labeled serving size,” and the food companies can continue to market the HFSS food by just reducing serving size. The marketing policy of the International Food and Beverage Alliance allows the signatories to develop their own nutrition criteria [69] which results in non-uniformity among the transnational member industries.
• The definition of child-directed marketing in these pledges (Table 4) is very lenient [39, 69, 71–73, 75, 77–80]. They restrict advertisements directed to children under 13 years of age with an audience share of 35% from this age group. Whereas, WHO [24], UNICEF [28], and the European Commission [23] clearly recommend the target audience for advertisement regulations to be <18 years of age.
• These pledges do not cover several important forms of food marketing communications. Licensed promotional characters are allowed to appear on food packaging and at point of sale including both end of aisle and shop checkouts. The brand marketing to children is also not restricted [78].

## Discussion

The earlier recommendations of WHO lacked universal nutrition criteria and suggested stepwise implementation and industrial self-regulation (Results Section A). Lack of nutrition criteria led some food industries to put forth misleading nutrition claims aimed at enhancing their product’s marketing. Nutrient profiling aims to produce coherent and consistent nutrition messages in the best interests of children. To date, all six of the regional offices of the WHO have developed a nutrient profiling model (Table 1), but only a few countries are following it. Statutory laws were found to follow the WHO nutrition criteria which is better than the self-regulations. For example, the nutrient profile developed by the WHO regional office of Europe [62] has only one category for cereals, and the permitted sugar level is 15 g/100 g. In contrast to that, the EU pledge nutrition criteria put cereals into different subcategories [40] with different thresholds allowing ready-to-eat breakfast cereals to market at the threshold of up to 27 g/100 g. According to Taillie et al. (2019) the nutrient criteria for restricting advertising can incentivize industries to reformulate their product [86]. Although reformulation can potentially benefit both industries and consumers by reducing unhealthy nutrients, the effectiveness of such incentives depends on rigorous enforcement and monitoring.

The gradual approach to implementing restrictions, where certain foods and forms of marketing are prioritized, has shown limited success. In some cases, countries could begin restrictions with a gradual approach of prioritizing some foods and forms of marketing over others, proceeding in various stages to an ultimate full-scale ban. However, the evolving evidence suggests that the gradual approach is paving the way for food industries to simply shift their advertisement from regulated to unregulated areas [86], which results in no improvement and leaves children inadequately protected [87]. For example, Industry-led voluntary pledges do not cover advertisements on the product packaging (Table 4). Hence, eye-catching cartoonish characters on the packaging of HFSS foods continue to act as silent salesman for food companies. Moreover, end-of-aisle and shop checkouts in the retail store also feature unhealthy foods targeted at children. This influences children’s practices both at the time of purchase and during consumption [88]. The flexible definition of child-directed marketing set by the industries (Results Section B) also allows them to shift HFSS food advertisement from children’s to non-children programs and prime-time family shows and children are reported to view 26% more ads on non-children’s TV programs [88]. Food industries direct their marketing on media and platforms for older kids which are highly viewed by the younger kids too [86]. Moreover, the threshold percentage of the audience that is children for the laws to apply is also set very high. For example, Ireland’s Children’s Commercial Communications Code [49] applies only if 50% or more audience is under 18 years of age. This percentage is hardly possible to reach in the high-income countries that have a low number of children.

Some regulations only cover certain forms of marketing communications and allow the industries to advertise their brand to children even when their products are not considered healthy by the nutrition criteria. For example, the Quick Service Restaurant Pledge in Australia does not allow fast food restaurants to advertise their menu to children unless they follow the nutrition criteria [78], but a commercial for the McDonald’s Happy Meal in Australia continued to be aired on children’s programs because it featured healthy items like milk and an apple slice along with HFSS foods in the same meal [89]. Contrary to that, a comprehensive approach of eliminating all forms of unhealthy food marketing to children of all ages through all marketing communication has the highest potential to obtain the desired outcome. Despite the clear evidence of the ineffectiveness of the gradual approach [28], to date, no country has adopted a comprehensive policy approach. There are diverse venues to target children, especially digital marketing on social media has become a cost-effective strategy since television is becoming less popular among the younger generation [90]. Therefore, the laws should also include new venues for food marketing.

Food and beverage industries have supported self-regulation and voluntary pledges due to their cost-effectiveness and flexibility compared to statutory laws. Self-regulation is often preferred because it reduces governmental oversight and judicial conflicts while allowing companies to maintain brand loyalty (Results Section C). However, the drawbacks of self-regulation include a lack of transparency, insufficient involvement of non-industrial stakeholders, and weak accountability mechanisms [91]. The limited effectiveness of voluntary pledges and the tendency for industries to prioritize their interests over public health suggest that statutory regulations may be necessary to achieve meaningful improvements in children’s nutrition. The evidence indicates that the benefits of comprehensive legislative measures outweigh the advantages of self-regulation, emphasizing the need for more robust and enforceable policy solutions at the level of statutory laws that must be prepared for the protection of both the food industry and its consumers alike. We do not urge a sudden ban on all the profitable businesses, but rather a state-level commitment to ensure that the food manufacturers produce and market responsibly while continuing to generate revenue for the governments.

### Limitations

The objective of this current review was to identify the food marketing policies and regulations aimed at mitigating childhood obesity. Consequently, the literature examined is restricted solely to childhood obesity, overlooking potentially valuable insights from food marketing policies addressing other adverse impacts on children. Similarly, childhood obesity is a multifaceted problem having a variety of risk factors, but we delimited our study to cover only the policies related to marketing and advertisement. The keywords used in the preliminary search to identify relevant policy databases were limited. However, they were only used for database identification and not the literature itself. Therefore, the review encompasses comprehensive literature from the selected databases. Furthermore, the review’s scope was constrained by the inclusion of only those documents that were available in the English language and the exclusion of non-English texts. However, to overcome this limitation, the data was triangulated from multiple websites and databases in an attempt to provide a holistic review. Additionally, peer-reviewed literature was omitted, and while evaluation studies could offer substantial evidence regarding the efficacy of policies and regulations, exploring this aspect fell beyond the scope of the present review. Therefore, this review presents the overall picture of policies while leaving a future direction for covering the implementation, efficacy, and feasibility of those policies in real settings. High-income countries have been the focus of this study, with potentially more robust implementation strategies. Conversely, low-income regions exhibit elevated rates of obesity yet may lack well-regulated implementation plans due to resource limitations. By comparing the implementation and effectiveness of policies, viable options can be identified for resource-constrained settings.

### Moving Forward

In order to address gaps in the regulatory framework, governments should clearly define marketing directed at children and establish criteria for classifying unhealthy food. Restriction on advertising should specifically target audiences under the age of 18, and efforts should be made to minimize the percentage of child viewers. It is advisable to regulate all forms of media communication aimed at children to prevent the food industry from shifting its marketing to unregulated platforms in order to circumvent legal regulations in place. Rather than vague and broad restrictions, legislation should target specific marketing techniques and apply to both traditional and modern methods. Governments around the globe should refrain from initiating or supporting self-regulatory efforts. Industry-led self-regulations and nutrition criteria should be avoided and WHO’s recommendations and nutrition profiling should be implemented.

### Conclusion

Global custodians of public health have provided clear guidelines and policy recommendations to address childhood obesity by regulating food marketing. Some high-income countries have partially implemented these policies and have restrictions on the marketing of unhealthy foods and beverages. It is essential to establish comprehensive statutory regulations that cover all forms of marketing and adapt to evolving media landscapes in order to effectively reduce children’s exposure to unhealthy foods and improve public health outcomes. Future efforts should concentrate on strengthening enforcement mechanisms to fully realize the potential of policies set out as early as the 1970s.

## Footnotes

1
http://www.greylit.org/

2
www.google.hu

## Abbreviations

BBB, Better Business Bureau; CRC, Convention on the Rights of the Child; CFBAI, Children’s Food and Beverage Advertising Initiative; EU, European Union; GINA, Global Database on the Implementation of Nutrition Action; HFSS, High in fat, free sugar, and salt; UNICEF, United Nations International Children’s Emergency Fund; UN, United Nations; WHA, World Health Assembly; WHO, World Health Organization.
