# Supplementary material for: Food Advertisement and Marketing Policies Aimed at Reducing Childhood Obesity: A Review of Existing Regulations in High-Income Countries
Source: Public Health Rev. 2024 Dec 20;45:1607103. doi: 10.3389/phrs.2024.1607103 (PMC11695118; doi:10.3389/phrs.2024.1607103)
Supplement: Supplementary file 1 [file DataSheet1.docx]

**Supplementary Material**

**Supplementary Figure S1. Framework of search strategy and literature selection adopted for review**

STEP 01- Initial search

Aim:

Identification of relevant grey literature sources

Sources:

1. Grey literature database (http://www.greylit.org/)
2. Google search engine (www.google.hu)

Key words:

“childhood”, “obesity”, “overweight”, “food”, “beverage”, “marketing”, “advertisement”, “laws”, “regulations”, “guidelines”, “recommendations”, “self-regulation”

Relevant grey literature sources identified:

1. Websites of international health agencies
2. University of Connecticut Rudd Center for Food Policy and Health, Pledge Database on Food Marketing to Children Worldwide
3. World Health Organization (WHO) Global Database on the Implementation of Nutrition Action (GINA)
4. World Cancer Research Fund NOURISHING policy database
5. Government websites for country specific statutory jurisdictions and self-regulation

STEP 02- Literature selection

Inclusion criteria:

- Full text documents
- Year of publication: 2000-2022
- English language
- Guidelines to prevent overweight and obesity through food & beverage advertisement and marketing

Exclusion criteria:

Documents highlighting:

- Marketing of breastmilk substitutes/ formula milk
- Alcoholic beverages
- School lunch program
- Exclusively one form of marketing
- Peer reviewed literature
- Newspaper/ magazine articles

STEP 03- Identification of regulations in High Income Countries

3a: Identification of high income countries:

Gross National Income per capita of $13,205 or more (World Bank, 2022)

N=82

3b: Search on:

University of Connecticut Rudd Center for Food Policy and Health, Pledge Database on Food Marketing to Children to identify authorities overseeing the food and beverage industries

3c: Search on:

GINA database to identify “voluntary codes or measures relevant to nutrition” and the “legislation relevant to nutrition

3e: Search on:

Govt. websites of selected countries n= 16 (having statutory laws or self-regulation of food marketing) for latest documents of the selected polices and laws

3d: Search on:

NOURISHING database for the policies of food environments that restrict food advertising and commercial promotion

**Supplementary Table S2.** **Food marketing guidelines of national agencies of United States. (Global, 2000-2022)**

| Authority | Title | Year | Target Audience | Recommendations | Implementation |
| --- | --- | --- | --- | --- | --- |
| American Psychology Association (APA) | Report of the APA Task Force on Advertising and Children (4) | 2004 | < 8 years of age | Restrict advertising during the children’s programs  Advertising disclaimers in children’s programs should be in their language and for the time length conducive to reading, hearing, and understanding  CARU should publicize the guidelines to parents so they can identify and report the deviation  Advertising in all forms should be prohibited in schools | Industrial self-regulation |
| National Academy of Medicine | Preventing Childhood Obesity: Health in the Balance (5) | 2005 | 2-18 years of age | Industries should develop and adhere to marketing guidelines | Industrial self-regulation |
| White House Task Force on Childhood Obesity | Solving the Problem of Childhood Obesity Within a generation (11) | 2010 | Not specified | Retailers should avoid instore marketing  Entertainment industry should limit the licensing of characters only to healthy food products  Food and entertainment industry should adopt the uniform nutritional standards for marketing  Industry should provide on air labelling to distinguish healthy food from unhealthy  If voluntary efforts are not useful FCC should revisit the rules | Collaboration between federal government and private industries for self-regulation |
| American Academy of Pediatrics (AAP) | Children, Adolescents, Obesity, and the Media [Policy Statement] (12) | 2011 | Not specified | Congress, FTC and FCC should ban the junk food advertisement during children program and payments for product placement in movie  Prohibit the interactive marketing of junk food through digital media  Restore power to FTC for tighter regulations | Statutory laws and mandatory implementation |
| Interagency Working Group (IWG) | Proposed Nutrition Principles to Guide Industry Self-Regulatory Efforts (10) | 2011 | 2-17 years of age; 30% for 2-11 years and 20% for 12-17 years | Foods marketed to children should contribute to the healthy diet and have minimum content of nutrients harmful for health and contribute in weight gain | Industrial self-regulation |

Every country in the world has ratified UN CRC except the United States (US). All food advertisement recommendations by the UN agencies are based on UN CRC, therefore it was necessary to review the stance of the federal bodies of the US regarding food advertisement to children. Guidelines and recommendations of national agencies of the US related to food and non-alcoholic beverage advertisement, childhood obesity and overweight between year 2000-2022 were retrieved through the websites of United States Department of Agriculture (USDA), Food and Drug Administration (FDA), Federal Trade Commission (FTC), Centers for Disease Control and Prevention (CDC), American Academy of Pediatrics (AAP), American Psychology Association (APA), and National Academy of Medicine (Table 2)

**Findings:**

Better Business Bureau (BBB) is a private, non-profit organization to advance marketplace trust. It established a self-regulatory program, Children’s Advertising Review Unit (CARU) back in the 1970’s and since then its guidelines are periodically reviewed (1). The focus of the CARU guidelines is to discourage unhealthy eating practices according to the USDA’s American dietary guidelines (2), with an emphasis on portion size depiction and difference between meal and snack in advertisement. The statutory law in this regard is the “Children’s Television Act”, which was passed in 1990 by the Federal Communication Commission (FCC). This act does not specify the rule of food advertisement except the prohibition of featured characters from children’s programs in commercial during that program and requires a clear separation between the program and commercials (3). The self-regulatory guidelines of CARU are more comprehensive than the Children’s Television Act and were initially praised by other national agencies, specially APA Task Force on Advertising and Children. But CARU operated on a complaint based system, so APA suggested that guidelines of CARU should be more publicized to better identify the deviations in marketing practices (4). APA recommended that as children are unable to differentiate between commercial and noncommercial content, the advertisement disclaimers during children’s programs should be in children’s language and the audio/video time should be long enough for them to easily understand it. Later, the Institute of Medicine also declared that industries should do more rigorous self-regulation and the advertisement must not violate the dietary guidelines developed by USDA and Department of Health and Human Services, and FTC should monitor compliance of industries (5).

In response to the multiple calls for industries to self-regulate, BBB established the Children’s Food and Beverage Advertising Initiative (CFBAI) in 2007. The initiative initially had 10 signatories but has now grown to 20 (6). The core principle of CFBAI includes the restriction of unhealthy food on both broadcast and non-broadcast media. CFBAI also developed its own Uniform Nutritional Criteria (7) that member companies must follow. But 85% of the food products that the CFBAI considers acceptable, could not be marketed to children according to the WHO Nutrient profile model (8). FTC also criticized that the nutrition criteria and the definition of children directed programs vary among the member companies and CFBAI should focus on the consistency of implementation (9).

In 2009, Interagency Working Group (IWG) was formed and had representatives from FTC, FDA, CDC and USDA to propose nutrition principles for self-regulation. IWG released its first and unfortunately the last comprehensive set of nutrition principles in 2010. IWG did not only set the age and audience share criteria for defining child directed advertisement but also provided quantitative limits of saturated and trans-fat, salt and added sugar in food marketing (10). However, these voluntary recommendations were abandoned as soon as they were proposed, due to extensive industrial lobbying. Keeping in mind the unfruitful efforts of BBB and IWG, the White House finally suggested that the FCC should revisit and modernize the laws to restrict food advertisement to children (11). AAP also urged congress to restore the power of the FTC for creating stricter regulations and FCC should ban all form of food advertisement to children (12).

**REFERENCES**

1. Children’s Advertising Review Unit, Council of Better Business Bureaus,. Self-Regulatory Program for Children’s Advertising. United States of America(2009). <https://bbbnp-bbbp-stf-use1-01.s3.amazonaws.com/docs/default-source/caru/caru_advertisingguidelines.pdf>

2. McGuire S. US department of agriculture and US department of health and human services, dietary guidelines for Americans, 2010. Washington, DC: US government printing office, January 2011. Adv. Nutr. (2011) 2(3):293-4. doi: 10.3945/an.111.000430.

3. Federal Communications Commission. Children's Television Act. United States of America(1990). <https://www.congress.gov/bill/101st-congress/house-bill/1677>

4. Kunkel D, Wilcox BL, Cantor J, Palmer E, Linn S, Dowrick P. Report of the APA task force on advertising and children. Washington, DC: American Psychological Association(2004). p. 60. <https://www.apa.org/pi/families/resources/advertising-children.pdf>

5. National Academies of Sciences Engineering and Medicine. Preventing Childhood Obesity: Health in the Balance. Washington, DC: The National Academies Press; (2005).

6. Council of Better Business Bureau. Children’s Food and Beverage Advertising Initiative (CFBAI). (2022). <https://bbbprograms.org/programs/all-programs/cfbai>

7. Council of Better Business Bureau, Children's Food and Beverage Advertising Initiative,. Category-Specific Uniform Nutrition Criteria. 2nd ed(2018). <https://bbbprograms.org/siteassets/documents/cfbai/cfbai-white-paper-final-pdf.pdf>

8. World Health Organization. WHO Regional Office for Europe nutrient profile model. (2015). <https://iris.who.int/bitstream/handle/10665/152779/WHO-EURO-2015-6894-46660-67850-eng.pdf?sequence=1>

9. Federal Trade Commission. Marketing Food to Children and Adolescents; A Review of Industry Expenditures, Activities, and Self-Regulation. (2008). <https://www.ftc.gov/sites/default/files/documents/reports/marketing-food-children-and-adolescents-review-industry-expenditures-activities-and-self-regulation/p064504foodmktingreport.pdf>

10. Interagency Working Group. Proposed Nutrition Principles to Guide Industry Self-Regulatory Efforts. United States of America(2011). <https://www.ftc.gov/sites/default/files/documents/public_events/food-marketed-children-forum-interagency-working-group-proposal/110428foodmarketproposedguide.pdf>

11. White House Task Force on Childhood Obesity. Solving the Problem of Childhood Obesity Within a generation. United States of America(2010). <https://letsmove.obamawhitehouse.archives.gov/white-house-task-force-childhood-obesity-report-president>

12. Strasburger VC, Communications Co, Media. Children, adolescents, obesity, and the media. American Academy of Pediatrics Elk Grove Village, IL, USA; (2011). p. 201-8.
